# Tribute to Dr. Judith G. Levin (1934–2023)

**DOI:** 10.3390/v16020178

**Published:** 2024-01-25

**Authors:** Karin Musier-Forsyth, Alan Rein, Eric O. Freed

**Affiliations:** 1 Department of Chemistry and Biochemistry, Center for Retrovirus Research, Center for RNA Biology, Ohio State University, Columbus, OH 43210, USA; 2 Retroviral Assembly Section, HIV Dynamics and Replication Program, Center for Cancer Research, National Cancer Institute, National Institutes of Health, Frederick, MD 21702, USA; reina@mail.nih.gov; 3 Virus-Cell Interaction Section, HIV Dynamics and Replication Program, Center for Cancer Research, National Cancer Institute, National Institutes of Health, Frederick, MD 21702, USA; efreed@mail.nih.gov


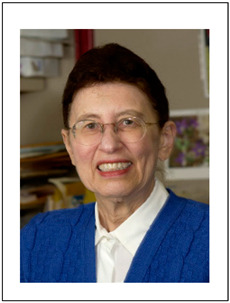


Dr. Judith G. Levin passed away in Teaneck, NJ, USA, on 8 December 2023. She was born to Harry and Ernestine Goldstein, public school teachers, in Brooklyn in 1934.

Dr. Levin attained a B.A. from Barnard College (majoring in chemistry), an M.A. from Harvard University (for a program in biochemistry), and a Ph.D. in biochemistry from Columbia University in the laboratory of David B. Sprinson. She was a Postdoctoral Fellow with Marshall Nirenberg at the National Heart Institute of the National Institutes of Health (NIH). In 1973, she joined the Laboratory of Molecular Genetics at the National Institute of Child Health and Human Development (NICHD) of the NIH, and in 1984, she became Head of the Unit on Viral Gene Regulation. Dr. Levin was the recipient of research funds from the NIH Intramural AIDS Targeted Antiviral Program from the inception of the program in 1987 until 2000. From 1992 until her retirement in 2014, she served as Head of the Section on Viral Gene Regulation in the Laboratory of Molecular Genetics and its successor program, the Program in Genomics of Differentiation in the Eunice Kennedy Shriver NICHD, NIH.

Dr. Levin’s Ph.D. work included studies on the enzymatic formation and isolation of 5-enolpyruvyl shikimate 3-phosphate, the target for the herbicide “Round-up” [1]. She joined the Nirenberg lab during what was arguably the most exciting time in genetics research in the 20th century and published several important papers on RNA codons and protein synthesis [2]. She was in the Nirenberg lab in 1968 when it was announced that he would share the Nobel Prize in Physiology or Medicine for “cracking the genetic code” and describing how it operates in protein synthesis.

In her early independent work, Dr. Levin studied murine leukemia virus (MLV) replication and made several groundbreaking discoveries. For example, she found that MLV virions can assemble in the absence of genomic RNA [3], which was later reported for other retroviruses including HIV-1. Her lab was also the first to report that MLV-infected cells contain two non-equilibrating pools of full-length viral RNA: one for encapsidation and the other functioning as the mRNA for the Gag precursor [4]. She subsequently published a series of elegant papers on the translational suppression of the MLV UAG termination codon coauthored by Alan Rein and Dolph Hatfield [5,6,7,8].

With the onset of the AIDS epidemic in the 1980s, Dr. Levin’s work shifted to mechanistic studies of HIV-1 replication. In collaboration with Eric Freed, she studied the effects of mutations in the HIV-1 capsid protein on infectivity, viral core architecture, and reverse transcription [9,10,11]. She also contributed significantly to research on the HIV-1 nucleocapsid protein (NC). In her first major paper published in the NC field, she showed, together with Louis Henderson, that HIV-1 NC plays a critical role in facilitating efficient and specific viral DNA synthesis by remodeling highly structured nucleic acid intermediates and blocking the formation of dead-end DNA self-priming products [12]. Her lab elucidated other key aspects of NC’s nucleic acid chaperone function, including the importance of the zinc-binding domains for helix-destabilizing activity, work conducted in collaboration with Henderson and Robert Gorelick [13]. She made many seminal contributions to this major field of retroviral research and, together with her collaborators, published highly cited reviews of the literature in this area [14,15,16].

More recently, Dr. Levin studied the human APOBEC3 (A3) proteins, a family of cellular cytidine deaminases that function as restriction factors and are part of the innate immune response to infection by HIV-1 and other viruses. Her lab was among the first to report biochemical studies of highly purified, catalytically active human A3G [17,18], and her group also contributed to important studies on A3A in collaboration with Angela Gronenborn [19,20].

In addition to her important scientific achievements, Dr. Levin was a wonderful role model for women scientists and an excellent mentor to all the students, postdocs, and research scientists who interacted with her. Her warmth, collegiality, and kindness stood out; she enjoyed scientific discussions as well as getting to know her collaborators and colleagues and hearing about their lives. Even in retirement, she remained connected to the field, attending conferences on zoom regularly and, most recently, co-editing (together with Alan Rein and Karin Musier-Forsyth) a 2023 Special Issue of *Viruses* entitled Molecular Genetics of Retrovirus Replication [21].

Beyond her love of science, she was dedicated to her family and Judaism. She is survived by her husband of 66 years, Jonathan Levin; her sons (and their wives), Joshua and Daniel (Dora and Risa, respectively); her grandchildren (Benjamin, Natan, Talia, and Esther); and her brother, Bernard Goldstein. She will be deeply missed by us and by all who knew her.
